# Shenxian-Shengmai Oral Liquid Improves Sinoatrial Node Dysfunction through the PKC/NOX-2 Signaling Pathway

**DOI:** 10.1155/2021/5572140

**Published:** 2021-04-10

**Authors:** Heng Zhang, Miao Hao, Lingkang Li, Keyan Chen, Jing Qi, Wei Chen, Xintong Cai, Chen Chen, Zhuang Liu, Ping Hou

**Affiliations:** ^1^Liaoning University of Traditional Chinese Medicine, Shenyang, China; ^2^Department of Laboratory Animal Science, China Medical University, Shenyang, China; ^3^Department of Cardiology, Affiliated Hospital of Liaoning University of Traditional Chinese Medicine, Shenyang, China

## Abstract

Sick sinus syndrome (SSS) is one of the common causes of cardiac syncope and sudden death; the occurrence of SSS is associated with the accumulation of ROS in the sinoatrial node (SAN). Shenxian-shengmai (SXSM) is a traditional Chinese medicine available as oral liquid that causes a significant increase in heart rate. The objective of this study is to observe the improvement of SXSM on SAN function in SSS mice and explore its potential mechanism. In the current study, SSS was simulated in mice by inducing SAN dysfunction using a micro-osmotic pump to inject angiotensin II (Ang II). The mouse model with SSS was used to determine the effect of SXSM on SAN function and to explore its potential mechanism. Furthermore, the HL-1 cell line, derived from mouse atrial myocytes, was used to simulate SAN pacemaker cells. Our results indicated that SXSM significantly increased the heart rate of SSS mice by reducing the AngII-induced accumulation of ROS in the SAN and by inhibiting the expression of HDAC4, thereby reducing the loss of HCN4, a critical component of the cardiac conduction system. MASSON staining revealed a reduction of SAN damage in SSS mice that were treated with SXSM compared with controls. In vitro experiments showed that AngII treatment caused an upregulation of the PKC/NOX-2 signaling pathway in HL-1 cells which could be prevented by pretreatment with SXSM. The protective effect of SXSM was attenuated upon treatment with the PCK agonist PMA. In conclusion, SXSM reduced the AngII-induced accumulation of ROS in the SAN through the PKC/NOX2 signaling pathway, improving the functioning of the SAN and preventing the decrease of heart rate in SSS mice.

## 1. Introduction

Sick sinus syndrome (SSS) is characterized by abnormal pacing or conduction dysfunction of the sinoatrial node (SAN) caused by pathological changes in the SAN and/or its surrounding tissues. This syndrome often manifests as various arrhythmias such as bradycardia, atrioventricular block, and sinus arrest. A common and serious disease, SSS, is one of the common causes of cardiogenic syncope and sudden death [1]. At present, the most effective treatment of SSS is artificial cardiac pacemaker implantation, but this method has several disadvantages including high costs, serious side effects, and many contraindications and complications. Moreover, the probability of atrial fibrillation after surgery is significantly increased [2, 3]. Conservative treatment of SSS mostly uses drugs such as atropine and isoproterenol. However, the use of these drugs is also associated with serious side effects including life-threatening malignant arrhythmias [4], and biological pacing is far from being used in clinical practice. Therefore, safe and effective drugs to treat SSS are urgently needed.

Shenxian-shengmai (SXSM) is a traditional Chinese medicine available as oral liquid that causes a significant increase in heart rate. Clinically, it is commonly used to treat bradyarrhythmia. Its main ingredients are ginseng, epimedium, psoralen, wolfberry, ephedra, asarum, salvia, and leech, and its exact curative effect and minor side effects make it a promising drug for the treatment of SSS [5–7].

Phase 4 automatic depolarization of pacemaker cells (P cells) is the basis for the autonomy of the SAN. It is currently believed that multiple ion currents may play a role in this process, including the activation of the funny current (*I*_f_), the decay of the potassium current, the continuous inward current, the TTX-sensitive background sodium current, and the calcium current. There has been an increasing interest in the role of *I*_f_ in 4-phase automatic depolarization [8]. As a cardiac pacing current, *I*_f_ is mainly produced by P cells in the SAN, which can cause the automatic depolarization of the autonomic cell action potential in phase 4 and plays a key role in the formation and maintenance of the heart rhythm [9]. The *I*_f_ channel is encoded by the hyperpolarized cyclic nucleotide cation-gated channel (HCN) gene family. The HCN family consists of 4 members: HCN1, HCN2, HCN3, and HCN4. Among these, HCN4 is the subtype with the highest expression in the SAN of various mammals, accounting for more than 80% of total HCN [10]. HCN4 is the main protein that constitutes the *I*_f_ channel, and it also plays a very important role in the development of the cardiac conduction system. HCN4 knock-out mice cannot survive [11], and the pacing frequency of mice with partial knockdown of HCN4 was significantly reduced [12]. A mouse model of cardiac HCN4 knockout showed severe bradycardia, atrioventricular block, and cardiac arrest [13]. Moreover, mutations in HCN4 were found to be associated with SSS in humans [14].

The expression of HCN4 in the cardiac conduction system is regulated by myocyte-specific enhancer-binding factor 2 (MEF2). The MEF2 transcriptional activator family consists of four members: MEF2A, MEF2B, MEF2C, and MEF2D. MEF2C is highly expressed in the cardiac system and plays an important role in heart development [15, 16]. Meanwhile, the activity of MEF2 is regulated by histone deacetylases (HDACs). Among them, only type II HDACs is expressed in the heart. HDAC4 is a type II HDAC that directly binds to MEF2C in a redox-dependent manner, silences its transcription, and inhibits the expression of HCN4. Since ROS plays a key role in SSS, inhibiting excessive oxidative stress and reducing ROS accumulation is a potential treatment target [17–19].

SXSM contains a variety of traditional Chinese medicines including ginseng, ephedra, and other ingredients that can significantly increase the heart rate and that have clear antioxidant effects [20–23]. However, it is currently unknown if SXSM could inhibit oxidative stress and improve SAN function in SSS. Therefore, this study aims to determine whether SXSM can increase the heart rate of SSS mice and to explore its mechanism to provide further evidence for the application of SXSM in SSS in humans. In this study, we used Ang II to induce SAN dysfunction in mice to simulate SSS in the presence or absence of intervention with SXSM. After identifying relevant blood indicators, the HL-1 cell line, derived from mouse atrial myocytes, was used to simulate P cells of the SAN to further study the mechanism of action of SXSM.

## 2. Methods and Materials

### 2.1. Animal Study

All mice were purchased from Liaoning Changsheng Biotechnology (China, Production License SCXK (Liao) 2018-0001) and were raised in a specific pathogen-free (SPF) animal laboratory with no restriction on diet. Fifteen C57B6 mice (male or female) were randomly divided into 3 groups: the sham surgery group (SHAM), the Ang II-induced treated SSS model group (SSS), and the SSS model group treated with SXSM group (SXSM) (*n* = 5 in each group). The mice were fasted for 24 hours without water following the method of Swaminathan et al. [24]. Subsequently, a subcutaneous micro-osmotic pump (Alzet model 1004, 0.11 ul/h, 28 d) was used to inject Ang II (3 mg/kg/d) or saline to induce SAN dysfunction to simulate SSS. The model preparation process was as follows: after the mouse neck skin preparation, anesthesia was done by intraperitoneal injection of 1% pentobarbital sodium (45 mg/kg). The mice were placed in a prone position fixed on the experimental animal operating table and an incision of about 1–1.5 cm was made in the neck to insert the capsule to which the assembled micro-osmotic pump was added. A small amount of gentamicin was applied to prevent infection and the incision was sutured. The SXSM group was given SXSM Oral Liquid (BUCHANG PHARMA, China) by gavage (5 ml/kg/d) on the second day after surgery. The SHAM group and SSS were given the same volume of saline. After 28 days, the heart rates of the mice in each group were recorded by electrocardiogram and the heart tissue was collected after euthanasia by intraperitoneal injection of sodium pentobarbital (150 mg/kg).

### 2.2. MASSON Staining

Collected heart tissues were cut into paraffin sections. The paraffin sections were deparaffinized and washed sequentially with distilled water. The nuclei were stained with Weigert's iron hematoxylin for 10 minutes. After washing the sections with distilled water, Masson's composite staining solution was applied for 10 minutes. The slices were soaked in a 2% (v/v) glacial acetic acid solution and then differentiated using a 1% dodeca molybdophosphoric acid solution for 5 minutes. Next, the sections were stained with aniline blue for 5 minutes without washing and then soaked in a 0.2% glacial acetic acid solution. Finally, the sections were dehydrated in 95% ethanol and absolute ethanol. Xylene was used as a clearing agent. Neutral glue was used for sealing.

### 2.3. Oxidative Stress Factor Assays

The blood of the mice was collected and the serum was obtained after centrifugation. The superoxide dismutase (SOD), catalase (CAT), and lipid peroxidation (MDA) assay kits were purchased from Solarbio Biotechnology (China).

### 2.4. Cell Culture

The HL-1 cell line that is derived from mouse atrial myocytes was purchased from ATCC (USA). Cells were seeded into cell culture dishes and cultured in DMEM/F12 (Sigma-Aldrich, USA) supplemented with 10% fetal bovine serum (Clark Bioscience, USA) and 1% penicillin/streptomycin (Solarbio Biotechnology, China). The cells were maintained in an incubator containing 5% CO_2_ at 37°C.

The cells were divided into 4 groups: CON, Ang II, SXSM, and SXSM + PMA. The CON group was the control group without intervention. The cells of the other groups were cultured for 12 hours and then treated with Ang II (Sigma-Aldrich, USA) for 12 hours. At the same time, SXSM was added to the cells of the SXSM group while SXSM and the PKC agonist PMA (Sigma-Aldrich, USA) were added to the SXSM + PMA group. The concentration of Ang II and PMA was 1 *μ*M and the concentration of SXSM oral liquid that did not result in a cytotoxic effect was as determined in preliminary experiments.

### 2.5. Cell Viability Assay

HL-1 cells (100 *μ*L) were seeded into 96-well plates at a density of 1 × 10^4^ cells per well. After incubation for 12 h, the cells were treated with various concentrations of SXSM (0.5 ml/L, 1 ml/L, 2 ml/L, 5 ml/L, and 10 ml/L) for 12 h, after which the cell counting kit-8 (CCK-8) reagent (APExBIO Technology, USA) was used to assay cell viability.

### 2.6. Immunofluorescence Staining

The paraffin sections of the SAN of mice from the three experimental groups were dewaxed and dehydrated for antigen retrieval. After blocking with 1% BSA for 1 h, the primary antibody was added, and the sections were placed in a humid box and incubated overnight at 4°C. After washing the sections, the fluorescein-labeled secondary antibody was added, followed by incubation at 37°C for 1 hour. After washing, an antifluorescence quenching mounting tablet containing DAPI was added, and the cells were observed under a fluorescence microscope. In vitro, the cells were seeded at 1 × 10^5^ cells in a cell culture dish for laser confocal microscopy. Following culture and treatment, the cells were fixed with 4% paraformaldehyde for 20 minutes and then permeabilized with immune permeabilization solution for 20 minutes. After washing with PBS, 1% BSA was added to block for 1 hour and then the primary antibody was added and the dishes were placed in a humid box and incubated overnight at 4°C. After washing, a fluorescein-labeled secondary antibody was added and incubated at 37°C for 1 h. After washing, an anti-fluorescence quenching mounting tablet containing DAPI was added and the cells were observed under a laser scanning confocal microscope. The HCN4 primary antibody was purchased from Santa Cruz Biotechnology (USA), and the NF160 primary antibody was purchased from Abcam (USA). The fluorescein-labeled secondary antibody was purchased from Proteintech (China).

### 2.7. ROS Assay: Dihydroethidium (DHE) and 2,7-Dichlorodihydrofluorescein Diacetate (DCFH-DA) Were Used

In vivo, heart tissues of mice were quickly frozen and then sliced. The DHE reagent was added to the slices and incubated at 37°C for 1 h. After washing with PBS, the SAN was observed with a fluorescence microscope.

In vitro, HL-1 cells were seeded at 1 × 10^5^ cells in a cell culture dish for laser confocal microscopy. After culture and treatment, DCFH-DA reagent was added and incubated at 37°C for 1 hour. After washing with PBS, the cells were observed using a confocal laser microscope. The DHE and DCFH-DA reagents were purchased from Beyotime Biotechnology (China).

### 2.8. Western Blotting

Proteins were separated by polyacrylamide gel electrophoresis in the presence of SDS. After electrophoresis in Tris-glycine buffer, the proteins were transferred to a polyvinylidene difluoride (PVDF) membrane. Next, the membrane was blocked in 5% skimmed milk in Tris-buffered saline containing 0.05% Tween-20 (TBST) for 1 h at room temperature. After washing with TBST, the membranes were incubated with primary antibody at 4°C overnight. After washing with TBST, the membrane was incubated with horseradish peroxidase- (HRP-) bound secondary antibody for 1 h at room temperature, then washed with TBST, and finally visualized using a chemiluminescence kit (Beyotime Biotechnology, China). The HDAC4 primary antibody was purchased from ABclonal Technology (China); the p47^phox^ primary antibody, NOX2 primary antibody, and HRP-bound secondary antibody were purchased from Proteintech (China).

### 2.9. Statistical Analysis

All experiments were repeated three times. The data were statistically analyzed using SPSS 17.0 software and expressed as mean ± SEM. Statistical comparison was carried out with one-way ANOVA. *p* < 0.05 was considered statistically significant (^*∗*^*p* < 0.05, ^*∗∗*^*p* < 0.01).

## 3. Results

### 3.1. SXSM Reduces Ang II-Induced SAN Dysfunction and Increases Heart Rate in SSS Mice

**Figure 1:**
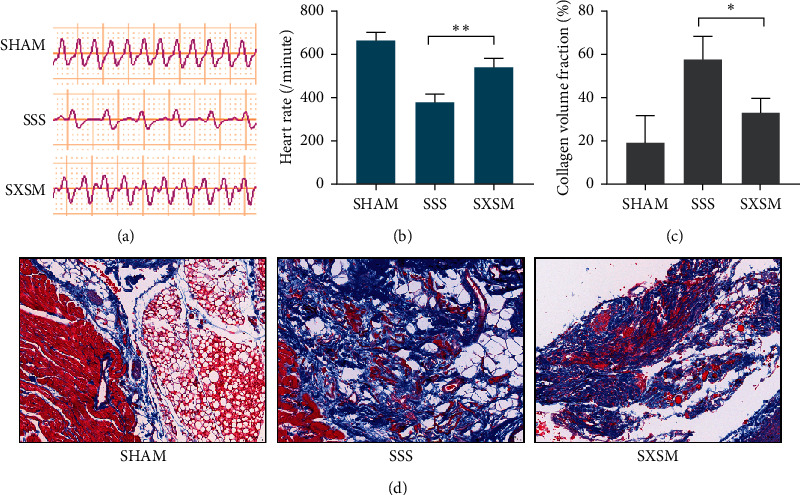
SXSM reduces Ang II-induced SAN dysfunction and increases heart rate in SSS mice. (a) Representative ECGs of mice in each group. (b) Mean heart rates in each group of mice. (c) Masson staining of the SAN of mice in each group. (d) Collagen volume fraction in the SAN of mice in each group. SHAM: mice with sham surgery; SSS: mice with Ang II treatment; SXSM: mice with Ang II and SXSM treatment; ^*∗*^*p* < 0.05, ^*∗∗*^*p* < 0.01. SAN, sinoatrial node; ECG, electrocardiogram; SXSM, Shenxian-shengmai oral liquid; SSS, sick sinus syndrome, Ang II, angiotensin II.

Heart rate and Masson staining were used to evaluate SAN dysfunction. The electrocardiogram (ECG) showed that the heart rate of mice in the SSS group was significantly lower than that of the SHAM group, while the heart rate of the SXSM group was significantly higher than that of the SSS group (Figures 1(a) and 1(b), ^*∗∗*^*p* < 0.01). Masson staining results showed that the degree of fibrosis of the SAN in the SSS group was significantly greater than that of the SHAM group, while the degree of fibrosis in the SXSM group had improved compared with the SSS group (Figure 1(c)). Analysis of the collagen volume fraction (CVF) showed that SXSM significantly reduced Ang II-induced SAN fibrosis (Figure 1(d), ^*∗*^*p* < 0.05). The assessment of the heart rate and Masson staining suggested that SXSM could significantly alleviate Ang II-induced SAN dysfunction in the SSS mice and prevent the decrease in heart rate.

### 3.2. SXSM Reduces ROS Accumulation in SAN and Oxidative Stress Factor in Serum

**Figure 2:**
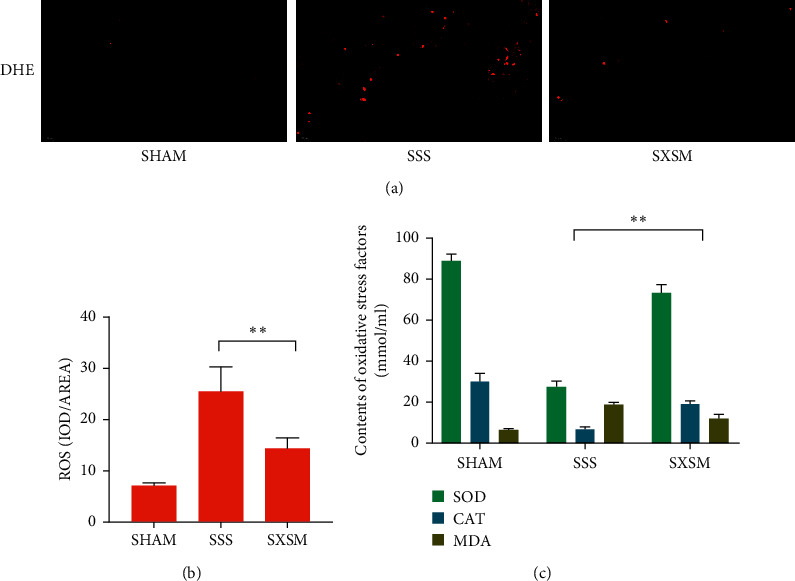
SXSM reduces ROS accumulation in the SAN and oxidative stress factors in serum of SSS mice. (a) Representative fluorescence images of ROS in the SAN of mice from each group. (b) ROS analysis using DHE. (c) Oxidative stress factors in serum of mice. SHAM: mice underwent sham surgery; SSS: mice underwent Ang II treatment; SXSM: mice underwent Ang II treatment in the presence of SXSM; ^*∗∗*^*p* < 0.01. SAN, sinoatrial node; SXSM, Shenxian-shengmai oral liquid; SSS, sick sinus syndrome, Ang II, angiotensin II; ROS, reactive oxygen species; DHE, dihydroethidium.

The ROS content in the SAN of mice was assayed using red fluorescent DHE. The stronger the red fluorescence intensity, the higher the ROS content. Fluorescence images showed that the fluorescence intensity in the SAN in the SHAM group was extremely low, while the fluorescence intensity in the SAN in the SSS group was high. In contrast, the fluorescence intensity in the SAN in the SXSM group was lower than that of the SSS group (Figure 2(a)). These results showed that the ROS content of the SAN in the SSS group was significantly increased compared with the SHAM group, while the ROS content of the SAN in the SXSM group was significantly lower than that of the SSS group (Figure 2(b), ^*∗∗*^*p* < 0.01). Meanwhile, the results of serum oxidative stress factors showed that, compared with the SHAM group, the SOD content and CAT content in the serum of mice in the SSS group were significantly reduced, while the MDA content increased, and that the oxidative stress factors of the SXSM group were significantly improved compared with the SSS group (Figure 2(c), ^*∗∗*^*p* < 0.01). These results indicated that SXSM could significantly prevent Ang II-induced peroxidative damage to the SAN in the SSS mice.

### 3.3. SXSM Significantly Increases HCN4 Expression in SAN of SSS Mice

**Figure 3:**
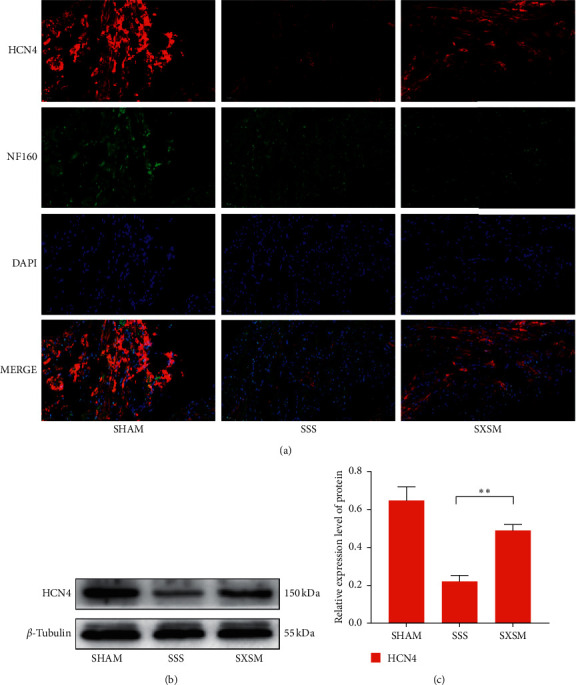
SXSM treatment prevents the reduction in HCN4 expression in the SAN of SSS mice. (a) HCN4/NF160 immunofluorescence staining of heart tissues of mice from the three groups. NF160 (green fluorescence) marks the location of the SAN. DAPI marks the location of the nuclei. (b) Western blot showing the expression of HCN4 in the SAN of mice from the different groups. *β*-tubulin was a loading control. (c) Quantitative analysis of the western blot results in (b). SHAM: mice underwent sham surgery; SSS: mice underwent Ang II treatment; SXSM: mice underwent Ang II treatment in the presence of SXSM; ^*∗∗*^*p* < 0.01. SAN, sinoatrial node; SXSM, Shenxian-shengmai oral liquid; SSS, sick sinus syndrome, Ang II, angiotensin II.

The expression of HCN4 is the basis for the *I*_f_ produced by the P cells. We used NF160 (green fluorescence) to mark the location of the SAN and detected the expression of HCN4 by red fluorescence. The immunofluorescence staining results showed ((Figure 3(a), top panels) that the expression of HCN4 in the SAN of the SSS group was significantly lower than that of the SHAM group, while the expression of HCN4 in the SAN of the SXSM group was significantly higher than that of the SSS group (Figure 3(a)). Western blot confirmed that differences in HCN4 expression between the groups were statistically significant (Figures 3(b) and 3(c), ^*∗∗*^*p* < 0.01). These results indicated that SXSM could effectively prevent the Ang II-induced loss of HCN4 in the SAN of SSS mice.

### 3.4. SXSM Reduces Ang II-Induced ROS Accumulation in HL-1 Cells

**Figure 4:**
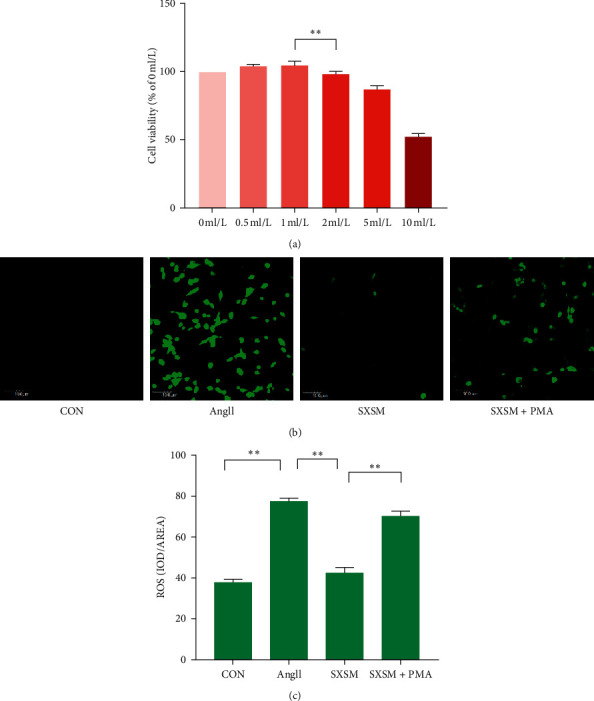
SXSM reduces Ang II-induced ROS accumulation in HL-1 cells. (a) A CCK-8 cell viability assay was used to determine the optimal concentration of SXSM. (b) ROS accumulation in HL-1 cells was assayed using green-fluorescent DCFH-DA. (c) ROS fluorescence photos statistical analysis. CON: control group cells; Ang II: cells treat with Ang II (1 *μ*M); SXSM: cells treated with Ang II and SXSM (1 ml/L); SXSM + PMA: cells treat with Ang II, SXSM (1 mL/L) and the PKC activator PMA (1 *μ*M); ^*∗∗*^*p* < 0.01. SAN, sinoatrial node; SXSM, Shenxian-shengmai oral liquid; SSS, sick sinus syndrome, Ang II, angiotensin II; ROS, reactive oxygen species.

To identify the optimal concentration of SXSM, we treated HL-1 cells with various concentrations of SXSM for 12h and then used the CCK-8 reagent to evaluate the cell viability of each group. No cytotoxicity was observed for SXSM at a concentration of up to 1 ml/L, while a slight cytotoxicity appeared when the concentration was 2 ml/L (Figure 4(a), ^*∗∗*^*p* < 0.01). Therefore, a concentration of SXSM of 1 ml/L was adopted in subsequent experiments. The ROS content of cells in each group was assayed using green-fluorescent DCFH-DA. The stronger the green fluorescence, the higher the ROS content. The Ang II group showed the highest cell fluorescence intensity, which was significantly reduced in the SXSM group. In contrast, fluorescence intensity was significantly higher in the presence of both SXSM and the PKC activator PMA compared with SXSM alone (Figure 4(b)). Our analysis showed that these differences in ROS content were statistically significant (Figure 4(c), ^*∗∗*^*p* < 0.01). These results showed that SXSM could significantly prevent Ang II-induced oxidative damage in HL-1 cells and reduce the accumulation of intracellular ROS, and this effect of SXSM could be offset by the addition of a PKC activator, suggesting that the antioxidant effects of SXSM were produced by inhibiting the activation of PKC.

### 3.5. SXSM Prevents Ang II-Induced HCN4 Loss in HL-1 Cells

**Figure 5:**
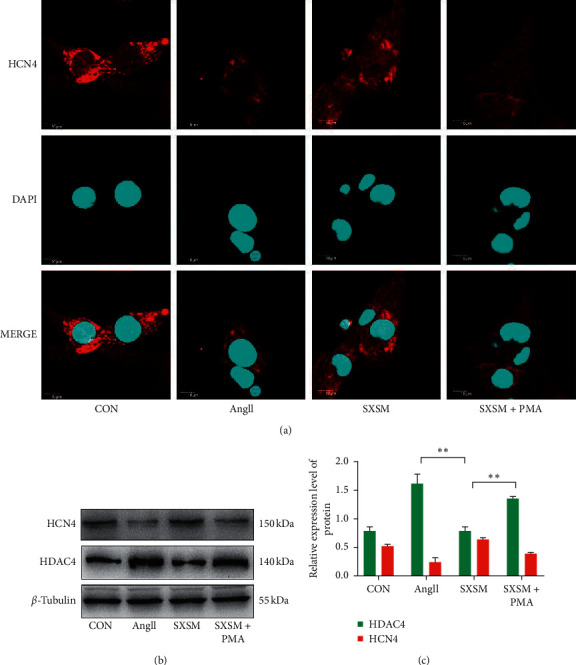
SXSM prevents Ang II-induced HCN4 loss in HL-1 cells. (a) Immunofluorescence staining of HCN4 (red). DAPI staining indicates the nuclei. (b) Western blot showing the expression of HCN4 and HDAC4. (c) Quantitative analysis of the western blot results in (b). CON: control group cells; Ang II: cells treated with Ang II(1 *μ*M); SXSM: cells treated with Ang II (1 *μ*M) and SXSM (1 mL/L); SXSM + PMA: cells treated with Ang II (1 *μ*M), SXSM (1 mL/L) and PMA (1 *μ*M); ^*∗∗*^*p* < 0.01. SXSM, Shenxian-shengmai oral liquid; Ang II, angiotensin II; PMA, PKC activator.

Immunofluorescence staining was used to detect the expression of HCN4 in HL-1 cells. The results showed that the HCN4 fluorescence intensity of HL-1 cells treated with Ang II was significantly reduced, while the HCN4 fluorescence of HL-1 cells treated with Ang II in the presence of SXSM was significantly increased compared with HL-1 cells treated with Ang II in the absence of SXSM. Importantly, the effect of SXSM was offset by PMA (Figure 5(a)). Western blot results showed that, compared with the untreated (CON) group, the expression of HDAC4 in the Ang II group was significantly increased, while the expression of HCN4 was significantly decreased. In the presence of both Ang II and SXSM, the expression of HDAC4 was significantly reduced and the expression of HCN4 was significantly increased compared with Ang II alone. The addition of PMA offset the effects of SXSM on the expression of HCN4 and HDAC4 (Figures 5(b) and 5(c), ^*∗∗*^*p* < 0.01). These results suggest that SXSM can significantly inhibit Ang II-induced ROS accumulation, thereby inhibiting the expression of oxidative stress-dependent HDAC4, and finally preventing the loss of HCN4, and this effect of SXSM can be offset by the PKC protein agonist PMA. Therefore, the effect of SXSM may be produced by inhibiting the activation of PKC.

### 3.6. Protein Assay of PKC/NOX2 Signaling Pathway

**Figure 6:**
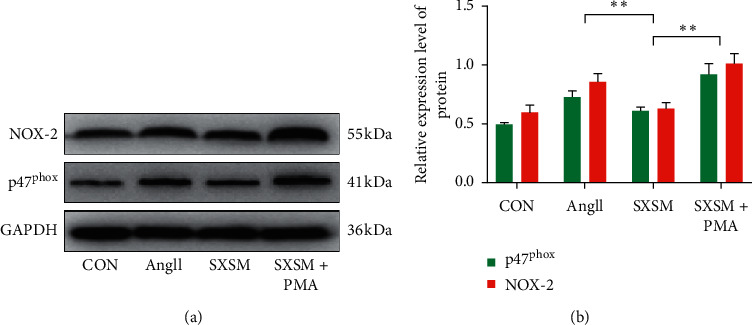
Protein assay of the PKC/NOX2 signaling pathway. (a) Western blot was used to assay the expression of NOX-2 and p47^phox^. GAPDH was used as a loading control. (b) Western blot quantitative analysis. CON: control group cells; Ang II: cells treated with Ang II (1 *μ*M); SXSM: cells treated with Ang II (1 *μ*M) and SXSM (1 mL/L); SXSM + PMA: cells treated with Ang II (1 *μ*M), SXSM (1 mL/L) and PMA (1 *μ*M); ^*∗∗*^*p* < 0.01. SXSM, Shenxian-shengmai oral liquid; Ang II, angiotensin II; PMA, PKC activator.

NOX-2 and p47^phox^ are critical components of the PKC/NOX-2 signaling pathway. We used western blotting to detect changes in the expression of NOX-2 and p47^phox^ on the membrane of each group of cells to evaluate the effect of SXSM on the signaling pathway. The results showed that the expression of NOX-2 and p47^phox^ was significantly increased after AngII treatment, suggesting that the PKC/NOX-2 signaling pathway of HL-1 cells was significantly activated. However, pretreatment of the HL-1 cells with SXSM significantly reduced the AngII-induced overexpression of NOX-2 and p47^phox^ while administration of the PKC agonist PMA canceled this effect of SXSM (Figure 6(a)). Quantitative analysis indicated that the differences in protein expression between the Ang II group and the SXSM group and between the SXSM group and the SXSM + PMA group were statistically significant (Figure 6(b), ^*∗∗*^*p* < 0.01). These results suggested that SXSM was able to inhibit the overactivation of the PKC/NOX2 signaling pathway induced by high levels of AngII, thereby producing a protective effect.

## 4. Discussion

Heart failure and high blood pressure are usually associated with SAN dysfunction [25, 26], which is characterized by excessive activation of renin-Ang II signaling and elevated ROS levels [27, 28]. In this study, we used a micro-osmotic pump containing Ang II to induce chronic SAN dysfunction to simulate SSS and found that Ang II can successfully induce fibrosis and dysfunction in the SAN of mice, which was expressed as a significant decrease in the heart rate. Moreover, we found that Ang II induced changes in oxidative stress factors in mice together with a significant accumulation of ROS in the SAN. These data suggest that our mouse model successfully mimics SSS in humans. Compared with the Ang II-treated mice in the SSS model group, administration of SXSM effectively alleviated the phenomenon of heart rate decline. Moreover, the mice in the SXSM group had significantly less fibrosis in the SAN compared with the SSS group and the changes in oxidative stress factors and ROS accumulation were also significantly less. These results suggested that SXSM can significantly prevent Ang II-induced SAN dysfunction. We therefore hypothesized that its mechanism may be related to the relief of Ang II-induced oxidative stress.

NADPH oxidase (NOX) plays an indispensable role in the balance of oxidative stress and is a key protein in the production of ROS. Among NOX family members, NOX-2 is distributed in large quantities in the heart and is the key source of ROS produced by cardiomyocytes [29]. Previous studies have shown that Ang II can activate p47^phox^ via the PKC signaling pathway and transport it to the membrane. This process activates the binding of p67^phox^ and NOX-2, thereby promoting the production of ROS [30–32].

To verify this mechanism, HL-1 cells which can stably express HCN4 and have detectable *I*_f_ [33] were used to simulate P cells. The results showed that after AngII treatment, the expression of NOX-2 and p47phox of HL-1 cells had increased which was associated with the accumulation of ROS. In addition, significant ROS accumulation was also observed in the SAN of SSS mice upon treatment with AngII, accompanying the increase in the degree of fibrosis in the SAN and the decrease in heart rate; these results suggest that the excessive activation of the PKC/NOX-2 signaling pathway induced by high levels of AngII may be the culprit that causes the accumulation of ROS and causes the damage to the SAN. Therefore, inhibiting the AngII-induced overactivation of the PKC/NOX-2 signaling pathway may be an important target for protecting and improving SAN function. We speculate that the mechanism of inhibition of Ang II-induced oxidative stress by SXSM may be related to its ability to inhibit the PKC/NOX-2 signaling pathway. Therefore, we administered SXSM while treating HL-1 cells with AngII and found that treatment with SXSM significantly reduced the accumulation of ROS induced by Ang II in HL-1 cells. Our western blot results confirmed that SXSM inhibited the expression of p47^phox^ and NOX-2, while the PKC inhibitor PMA canceled the effect of SXSM. We therefore speculate that SXSM inhibits Ang II-induced oxidative stress through the PKC/NOX-2 signaling pathway.

HCN4 is the material basis for the generation of *I*_f_ and maintaining sinus rhythm. Our study found that treatment with Ang II can induce the loss of HCN4 while inducing the accumulation of ROS in the SAN of mice, while SXSM restored the expression of HCN4 while inhibiting the production of ROS. Our study also found that treatment with PMA canceled the effect of SXSM and induced the ROS accumulation in HL-1 cells. Furthermore, the expression of HCN4 was also reduced, and the expression of HDAC4 was increased. Therefore, we believe that Ang II induced oxidative stress through the PKC/NOX-2 pathway, which caused the accumulation of ROS and increased the expression of HDAC4, which in turn reduced the expression of HCN4 by inhibiting MEF2C, ultimately causing a decrease in heart rate. SXSM may reduce the accumulation of ROS by inhibiting the PKC/NOX-2 signaling pathway, which in turn inhibits the loss of HCN4 and prevents the decrease in heart rate.

In this study, we found that SXSM can significantly inhibit Ang II-induced SAN dysfunction and improve heart rate in a mouse model of SSS. Our results furthermore indicate that the mechanism may be related to the PKC/NOX-2 signaling pathway. These studies provide new evidence that SXSM can be used for the clinical treatment of SSS. SXSM, a traditional Chinese medicine oral liquid, is a complex mixture of many components. In the future, we plan to use bioinformatics analysis technology to carry out the next step of our research, namely, to determine the effect of isolated, purified components of SXSM in SSS, and to increase its efficiency and reduce the toxicity of SXSM.

## Data Availability

The data used to support the findings of this study are available from the corresponding author upon request.
